# Supplementary material for: Factors for arboviral seropositivity in children in Teso South Sub County, Kenya
Source: PLoS One. 2025 Oct 14;20(10):e0328944. doi: 10.1371/journal.pone.0328944 (PMC12520415; doi:10.1371/journal.pone.0328944)
Supplement: Attachment — Submitted filename: Response to the Editors_PONE-D-25-31972-Rev2 - 24082025.pdf [file pone.0328944.s003.pdf]

Response to Reviewers' - PONE-D-25-31972_Revision2

Dear Editors,

We appreciate your review of our revised manuscript and request. We have adressed your request indicated in italics under each request.

PONE-D-25-31972R1

Factors for arboviral seropositivity in children in Teso South Sub County, Kenya
Dr Mary Inziani

Dear Dr. Inziani,

We've checked your submission and before we can proceed, we need you to address the following issues:

1. In the online submission form, you indicated that [Data cannot be shared publicly because they include clinical data with patient identifiers. Data are available from the Director General, Kenya Medical Research Institute (director@kemri.go.ke) for researchers who meet the criteria for access to confidential data.].

All PLOS journals now require all data underlying the findings described in their manuscript to be freely available to other researchers, either
1. In a public repository,
2. Within the manuscript itself, or
3. Uploaded as supplementary information.

This policy applies to all data except where public deposition would breach compliance with the protocol approved by your research ethics board. If your data cannot be made publicly available for ethical or legal reasons (e.g., public availability would compromise patient privacy), please explain your reasons on resubmission and your exemption request will be escalated for approval."

Response:

Thank you. We have removed identifiers from the study data base. We have provided the dataset as supplementary information - Supplementary Table 1 (S1_Table.xlsx).

We have edited the results section and cited the supporting information in the manuscript text, in the Results section, lines 159-167. We have also indicated the Supplementary information at the end of the manuscript (Lines 648-650).

We've returned your manuscript to your account. Please resolve these issues and resubmit your manuscript within 21 days. If you need more time, please email the journal office at plosone@plos.org. We are happy to grant extensions of up to one month past this due date. If we do not hear from you within 21 days, we will withdraw your manuscript.

Response:

We have resolved the issue raised and resubmitted the revised manuscript.

Please log on to PLOS Editorial Manager at https://www.editorialmanager.com/pone/ to access your manuscript. You will find your manuscript in the 'Submissions Sent Back to Author' link under the New Submissions menu. Be sure to remove your previous manuscript file if you are uploading a new file in response to these requests. After you've made the changes requested above, please be sure to view and approve the revised PDF after rebuilding the PDF to complete the resubmission process.

We are requesting these changes to comply with the PLOS ONE submission guidelines (https://journals.plos.org/plosone/s/submission-guidelines). Please note that we won't send your manuscript for review until you have resolved the above requests.

Thank you for submitting your work to PLOS ONE and supporting our mission of Open Science.

Kind regards,

Adrian Cyrus Luczon

PLOS ONE
