# Supplementary material for: Factors for arboviral seropositivity in children in Teso South Sub County, Kenya
Source: PLoS One. 2025 Oct 14;20(10):e0328944. doi: 10.1371/journal.pone.0328944 (PMC12520415; doi:10.1371/journal.pone.0328944)
Supplement: Attachment — Submitted filename: Responses to PNTD-D-23-00424 Review Comments.docx [file pone.0328944.s002.docx]

Dear Dr Muyeku,

Thank you very much for submitting your manuscript "Risk factors for arboviral seropositivity in children in Teso South Sub County, Western Kenya" (PNTD-D-23-00424) for consideration at PLOS Neglected Tropical Diseases. As with all papers peer reviewed by the journal, your manuscript was reviewed by members of the editorial board and by several independent peer reviewers. Based on the reports, we regret to inform you that we will not be pursuing this manuscript for publication at PLOS Neglected Tropical Diseases.

The reviews are attached below this email, and we hope you will find them helpful if you decide to revise the manuscript for submission elsewhere. We are sorry that we cannot be more positive on this occasion. We very much appreciate your wish to present your work in one of PLOS's Open Access publications. 

Thank you for your support, and we hope that you will consider PLOS Neglected Tropical Diseases for other submissions in the future.

Sincerely,

Salorme Adukpo
Guest Editor
PLOS Neglected Tropical Diseases

Abdallah Samy
Section Editor
PLOS Neglected Tropical Diseases

Shaden Kamhawi
co-Editor-in-Chief
PLOS Neglected Tropical Diseases
orcid.org/0000-0003-4304-636XX

Paul Brindley
co-Editor-in-Chief
PLOS Neglected Tropical Diseases
orcid.org/0000-0003-1765-0002


**************************************
Reviewer's Responses to Questions

Key Review Criteria Required for Acceptance?
As you describe the new analyses required for acceptance, please consider the following:

Methods
-Are the objectives of the study clearly articulated with a clear testable hypothesis stated?
-Is the study design appropriate to address the stated objectives?
-Is the population clearly described and appropriate for the hypothesis being tested?
-Is the sample size sufficient to ensure adequate power to address the hypothesis being tested?
-Were correct statistical analysis used to support conclusions?
-Are there concerns about ethical or regulatory requirements being met?

Reviewer #1: -Are the objectives of the study clearly articulated with a clear testable hypothesis stated? Yes, but no limitations are discussed including cross-reaction among related viruses.

**A ”Study Limitations” section has been added, addressing crossreactivity in addition to other limitations.**

-Is the study design appropriate to address the stated objectives? Yes, but no mention of the differences in IgG vs. IgM are included.

**The study limitations section has also addressed this. In a previous manuscript, the differences in IgG and IgM were addressed. Please see Inziani M, Adungo F, Awando J, Kihoro R, Inoue S, Morita K, et al. Seroprevalence of yellow fever, dengue, West Nile and chikungunya viruses in children in Teso South Sub-County, Western Kenya. Int J Infect Dis. 2020;91:104-10.**

-Is the population clearly described and appropriate for the hypothesis being tested? No. It is difficult to tell the age range of the children and the extent from each site and why different numbers of kids were tested for different viruses.

**The population description has been presented as Table 1.**

**The section on study settings, site and population has been completely rewritten to address these concerns.**

-Is the sample size sufficient to ensure adequate power to address the hypothesis being tested? Yes

-Were correct statistical analysis used to support conclusions? I don't think corrections were made for multiple comparisons.

**Both univariate and multivariate analysis have been done comprehensively and presented, as described from lines 130-147**

-Are there concerns about ethical or regulatory requirements being met? No, but they say that some of the testing was previosuly published in ref #13- De Rivera IL, Parham L, Murillo W, Moncada W, Vazquez S. Humoral immune response of dengue hemorrhagic fever cases in children from Tegucigalpa, Honduras. The American journal of tropical medicine and hygiene. 2008;79(2):262-6. which doesn't make any sense.

**Participant characteristics have been previously reported in Inziani M, Adungo F, Awando J, Kihoro R, Inoue S, Morita K, et al. Seroprevalence of yellow fever, dengue, West Nile and chikungunya viruses in children in Teso South Sub-County, Western Kenya. Int J Infect Dis. 2020;91:104-10.**

Reviewer #2: The methods are are well written. The study objectives are clear. The study design is appropriate. The study population is clearly described and the sample size sufficient and ensures adequate power for the study hypothesis. The statistical analysis supports some of the conclusions. The ethical requirement of the study were met.

Reviewer #3: The objectives of this study are clearly articulated;  the population is clearly described and appropriate for this study; the sample size is sufficient to ensure adequate power to address this hypothesis. But ethically, I find that the parental consent for children under 7 years was not asked.

**Parental consent for children below seven years of age was sought in all instances. The ethical considerations section has been revised to reflect this. Please see line 153.**

--------------------

Results
-Does the analysis presented match the analysis plan?
-Are the results clearly and completely presented?
-Are the figures (Tables, Images) of sufficient quality for clarity?

Reviewer #1: -Does the analysis presented match the analysis plan? Yes but it is insufficient given flavivirus cross reactivity.

**The analysis was redone comprehensively; additionaly, crossreactivity has been pointed out as a study limitation.**

-Are the results clearly and completely presented? No. Only very large statistical tables are included.

**All the results and figures tables been redone. The tables are mush simpler and ther statistical analysis simplified.**

-Are the figures (Tables, Images) of sufficient quality for clarity? No.

**All the results and figures tables been redone to simplify and enhance clarity.**

Reviewer #2: The analysis matches the analysis plan. 
Some of the results are clearly presented, but not completely.

**The statistical analysis has been redone and the results have been presented differently to enhance clarity.**

The tables are clearly presented.

Reviewer #3: The results are presented in a partially clear manner. There are some results that require clarification: 
1.      Did you measure the distance between houses and vegetation to qualify as near and far? give details.

**Guardians were asked if there were water bodies (including Rivers, Swamps, Ponds, Canals or Lakes near the house. The distance was not measured, but if they responded as yes, they were asked to estimate the distance from the house.**

2.      You say that you used yellow fever vaccination books for those who had them. Did you consider this entity in the analyses? and how many?

**Our preliminary analysis included yellow fever vaccination books. Only 11 such books were seen. In the end, the data had no significant impact on prevalence in any analysis.**

3.      Which sex influences seropositivity?

**Female children had a significantly higher prevalence. Please see Table 2.**

4.      Instead of saying "being a woman increases the risk...", replace with "being a female child...".

**The revised manuscript has resolved this.**

5.      Instead of "the elderly", use "children aged 6 to 9".

**The revised manuscript has resolved this.**

6.      I find a contradiction when you speak of a decreased risk in children aged 6 to 9 in multivariate and a high risk in univariate?

**This was an error on our part due to inappropriate analysis. In the end, it is clear that older children had higher prevalences in either the univariate or multivariate models.**

Are the figures (tables, images) of sufficient quality to be clear?

--------------------

Conclusions
-Are the conclusions supported by the data presented?
-Are the limitations of analysis clearly described?
-Do the authors discuss how these data can be helpful to advance our understanding of the topic under study?
-Is public health relevance addressed?

Reviewer #1: -Are the conclusions supported by the data presented? Not exactly given no distinction between IgG and M, cross-reactivity and multiple comparisons.

**In a previous manuscript, the differences in IgG and IgM were addressed. Please see Inziani M, Adungo F, Awando J, Kihoro R, Inoue S, Morita K, et al. Seroprevalence of yellow fever, dengue, West Nile and chikungunya viruses in children in Teso South Sub-County, Western Kenya. Int J Infect Dis. 2020;91:104-10.**

**In this manuscript, we have discussed the limitations around crossreactivity.**

-Are the limitations of analysis clearly described? Not listed at all.

**A “Study Limitations” section has been added**.

-Do the authors discuss how these data can be helpful to advance our understanding of the topic under study? Some suggestions of public health relevance are listed.

**The public health relevance has been rewritten to strengthen it.**

-Is public health relevance addressed? yes but an exhaustive list of all the previously work in Kenya, particularly western Kenya is not included.

**Additonal publications indicating previous work in Kenya have been referenced.**

Reviewer #2: The conclusions can be presented clearer than they are now.
The study limitations of analysis are not that clear. in the discussion.
The explanation can be done better.

**The conclusions section has been revised to improve it.**

The public health relevance is addressed.

Reviewer #3: The conclusions are clear in relation to the results, but the limitations of the analysis are not clearly described, especially for clinical elements pointing to arbovirus infections. These elements have not been documented. 

**A “Study Limitations” section has been added.**

The authors clearly discuss how these data can be useful in public health

--------------------

Editorial and Data Presentation Modifications?
Use  this section for editorial suggestions as well as relatively minor modifications of existing data that would enhance clarity. If the only modifications needed are minor and/or editorial, you may wish to recommend “Minor Revision” or “Accept”. 

Reviewer #1: (No Response)

Reviewer #2: The study  write up can be improved as follows. Could the authors add a figure that shows clearly the results from the indirect ELISA tests they performed for the viruses they tested for. Most of the results they present are the statistical analysis they performed.  It would enhance their presentation if they also show how the study participants numbers relate to the different risks factors they present in the various tables. The authors show rewrite their conclusions as what is presented now are general outcomes from their studies.

**To address these concerns, Table 1 has been added to present the various characteristics.**

Reviewer #3: Minor Revision

--------------------

Summary and General Comments
Use  this section to provide overall comments, discuss strengths/weaknesses of the study, novelty, significance, general execution and scholarship. You may also include additional comments for the author, including concerns about dual publication, research ethics, or publication ethics. If requesting major revision, please articulate the new experiments that are needed.

Reviewer #1: 1. The first sentence in intro needs to be clarified as it portrays that the arboviruses listed can all be spread by Culex and Anopheles mosquitoes. Only WNV is spread by Culex- all the others listed are vectored by Aedes in Africa. Reference to Anopheles should be deleted.
**This has been revised. It was actually somewhat misleading. We apologise.**

2.  Many of these arboviruses (DENV, WNV, and YFV) cross-react, so using ELISA alone makes it  difficult to distinguish flaviviruses from one another. Also, because many of the viruses are vectored by the same mosquito- I wouldn’t imagine that RFs would differ between viruses. Mosquito nets should not have much of an effect as well because many of these are day biting mosquitoes.

**This is actually intuitive. That said, we have discussed the perceived differences at some length in the manuscript.**

3. Why were those age cut offs used to categorize the children? What were the ages of the youngest seropositive kids for each virus?

**Children were categorised through school going behaviour. For instance, 1-3 year old children tend to be found at home, 3-6 at preschool and 6 and above in primary school. We can show that school going behaviour and exposure are related.**

4. Were multiple comparisons accounted for in the analysis?

**Yes. In the revised manuscript both univariate and multivariate analysis have been presented.**

5. The authors did not include substantial work done in this area by others. Several important papers describing seroepidemiology in Western Kenya are not included but should be and are listed here:
1. Sutherland, L. J., et al (2011).  Serological evidence of arboviral infections among humans in Kenya. American Journal of Tropical Medicine & Hygiene, 85(1), 158-61. PMC: 3122361. doi: 10.4269/ajtmh.2011.10-0203
2. Vu, D. M.et al (2017). Unrecognized dengue virus infections in children, western Kenya, 2014-2015. Emerg Infect Dis., 23(11), 1915-1917. doi: 10.3201/eid2311.170807.

At other sites in Kenya:At other sites in Kenya:
1.      LaBeaud, A. D. et al (2015). High rates of O'nyong Oyong and Chikungunya virus transmission in coastal Kenya. PLoS Negl Trop Dis, 9(2). doi:10.1371/journal.pntd.0003436. PMC: 4319898.
2.      Vu, D., et al. (2017). Dengue and West Nile virus transmission in children and adults in coastal Kenya. AJTMH, 96(1), 141-143. doi: 10.4269/ajtmh.16-0562. PMID: 27821697. PMC: 5239681.
3.      Grossi-Soyster, E. N., et al (2017). Serological and spatial analysis of alphavirus and flavivirus prevalence and risk factors in a rural community in western Kenya. PLoS Negl Trop Dis., 11(10) e0005998. doi: 10.1371/journal.pntd.0005998.
4.      Shah, M. M., et al (2020). High dengue burden and circulation of 4 virus serotypes among children with undifferentiated fever in Kenya, 2014-2017. Emerging Infectious Diseases, 26(11), 2638-50. https://dx.doi.org/10.3201/eid2611.200960

**These references have now been presented in the revised manuscript.**

6. What were the different seroprevalence rates for IgM vs IgG in the studied population? I am unsure how to relate any of the clinical data to the seroprevalence because I can’t tell what is acute vs remote exposure. There needs to be an overall table that describes the kids in total and those positive for each virus.

**The assay employed did not differentiate between the specific IgA, IgM or IgG antibodies.**

**To address these concerns describing the children in total, Table 1 has been added to present the various characteristics.**

7. For this paragraph in discussion: When we looked at preschool, lower primary and upper primary groups, we found that the upper primary group had a significantly increased risk of arbovirus seropositivity attributable to WNV. However, in the multivariate model, each group had a significantly decreased risk of seropositivity to an arbovirus, mainly attributable to CHIKV and WNV. This finding has baffled us, and we posit that there is an additional variable that may explain this finding.
Perhaps it is related to age?

**Reanalysis has indicated that this interpretation was erroneous. Culex mosquitoes are important, and age is related to exposure directly.**

8. Dengue, chikungunya and yellow fever are not capitalized. Correct throughout.

**These corrections have been done.**

Reviewer #2: The study looks at risk factors of arboviral seropositivity in Kenyan children. The methods for the study are well written, however the results section could be enhanced to make the statistical analysis presented much clearer.

**The statistical analysis has been redone and the results section revised comprehensively.**

The discussion done should include the study limitations as there are several from the design of the experiments done by the authors. the authors should also improve their conclusions with actual findings from the study rather than the general statements they have written in their conclusion section.

**The conclusions section has been revised in line with the study findings.**

Reviewer #3: 1. the strengths of this study lie in its originality and its interest in public health, especially the study of those risk factors that would have an impact on arbovirus transmission.
2. the study's weaknesses lie in the lack of in-depth analysis of the clinical signs that would point to the diagnosis of arboviruses.

**This weakness has been described in the study limitations.**

3. The author did not provide parental consent for children under 7 years of age. Was the children's consent written or verbal? And if verbal, how can we confirm how it was documented and attested?

**The parental consent for children below 7 was sought and was written. Please see the ethical statement.**

4. In the title, it would be better to remove "clinical factors".

**This has been done.**

--------------------

PLOS authors have the option to publish the peer review history of their article
